# Primary cilium disassembly: from mechanisms to roles in physiology and disease

**DOI:** 10.1242/jcs.264820

**Published:** 2026-06-30

**Authors:** Carolyn M. Ott, Jennifer Lippincott-Schwartz, David K. Breslow

**Affiliations:** 1. Janelia Research Campus, Howard Hughes Medical Institute, Ashburn VA 20147, USA; 2. Department of Molecular, Cellular, and Developmental Biology, Yale University, New Haven, CT 06511, USA; 3. Wu Tsai Institute, Yale University, New Haven, CT 06510, USA

**Keywords:** primary cilia, cilia disassembly, ciliopathy, ciliogenesis, basal body, centrosome

## Abstract

Primary cilia are micron-scale cellular compartments with key roles in cell signaling and disease. Cilium assembly has been delineated as a multi-step process that is disrupted in ciliopathies, yet many features of cilia disassembly remain incompletely understood. Here, we review recent insights into cilia disassembly. In addition to discussing the longstanding link between cilia disassembly and the cell cycle, we consider new findings that broaden our understanding of the molecular mechanisms and physiologic contexts that govern cilia disassembly. We also synthesize recent evidence that mutual antagonism between assembly and disassembly pathways governs ciliation state. Together, these studies reveal that both excessive and impaired disassembly can perturb ciliary signaling and function, with consequences for development, tissue homeostasis and tumorigenesis. Motivated by these advances, we propose a new ciliopathy subclassification – disassembly-related ciliopathies – to encompass diseases in which aberrant cilia disassembly contributes to pathogenesis. Continued exploration of the mechanisms controlling cilia disassembly will provide both fundamental biological insights and open new avenues for targeted treatment of diseases caused by ciliary dysfunction.

## Summary statement

This Review explains how recent advances have expanded our models of how, when and where primary cilia disassemble and discusses emerging roles of altered disassembly in disease.

## Introduction

Cells regulate organelle turnover to maintain cellular functions and adapt to external cues. Unlike most organelles, only one primary cilium exists per cell, and its presence or absence is dynamically regulated. Cilia are also distinctive because they are part of the cell surface, enabling them to sense changes in the extracellular environment and relay signals to the cell body (Hilgendorf et al., 2024; Mill et al., 2023; Ott and Lippincott-Schwartz, 2025). As signaling centers, primary cilia are required for mammalian embryonic development (Derderian et al., 2023; Mill et al., 2023; Reiter and Leroux, 2017). Cilia biogenesis and disassembly are therefore tightly regulated, and timely disassembly is important for cell fate transitions, signaling suppression and adaptation to physiological stimuli (Breslow and Holland, 2019; Gopalakrishnan et al., 2023; Ho and Stearns, 2021; Ott and Mukhopadhyay, 2025; Sanchez and Dynlacht, 2016).

**Figure 1:**
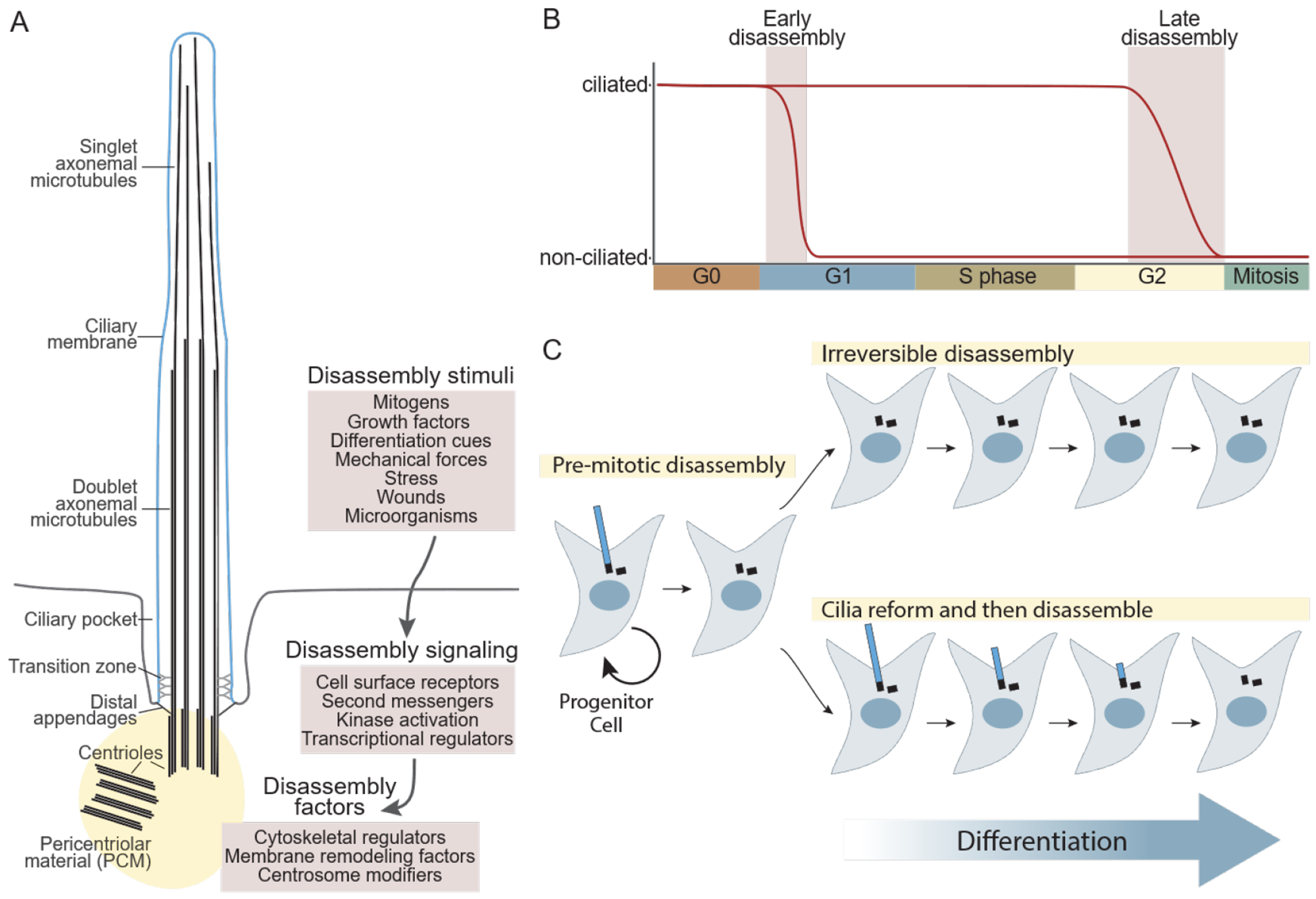
Physiologic contexts of cilia disassembly (A) Key structural features of primary cilia are illustrated, along with a general scheme for how disassembly stimuli initiate cilia disassembly. These stimuli activate signaling pathways that engage disassembly factors with complementary mechanistic roles. (B) Cilia disassembly occurs prior to mitosis. Early disassembly occurs in G1; late disassembly occurs in G2. In some cell populations, disassembly can occur at either time. (C) Cilia can be lost during differentiation. This loss can occur through pre-mitotic disassembly followed by repression of cilia assembly after mitosis (top) or by disassembly independent of mitosis (bottom).

Primary cilia are small but intricate cellular structures that are evolutionarily related to motile cilia and flagella. The core of the cilium is the axoneme, comprised of nine doublet microtubules that extend directly from the mother centriole (Fig. 1A). The mother centriole thus anchors the cilium at its base and is referred to as the basal body when templating a cilium (Breslow and Holland, 2019; Sanchez and Dynlacht, 2016). The ciliary membrane surrounds the axoneme and is anchored to the centriole distal appendages (Kumar and Reiter, 2021; Ma et al., 2023). Just above the distal appendages, the transition zone creates a selective filter that compartmentalizes the cilium from the cell body, thereby allowing cilia to have a distinct protein and lipid composition (Garcia-Gonzalo and Reiter, 2017; Mercey et al., 2024; Nachury and Mick, 2019; Reiter and Leroux, 2017; Rohatgi and Snell, 2010; Schermer et al., 2025). In some cells the basal body is recessed, forming a membrane invagination called the ciliary pocket (Benmerah, 2013; Rohatgi and Snell, 2010). Centriolar and ciliary microtubules have specialized architecture and extensive post-translational modifications (PTMs) and are among the most stable microtubules in the cell, able to withstand physical stresses and sustain function across decades in long-lived cells (Arrojo et al., 2019; Banterle and Gonczy, 2017; Behnke and Forer, 1967; Biven and Wang, 2025; Saunders et al., 2025). Removal of cilia therefore requires a dedicated process (Breslow and Holland, 2019; Mirvis et al., 2019; Sanchez and Dynlacht, 2016).

Cilia have long been known to disassemble prior to mitosis. Foundational studies in cultured mammalian cells established that serum mitogens induce quiescent cells to disassemble their cilia (Tucker et al., 1979a; Tucker et al., 1979b) and that the cell cycle-regulatory kinase Aurora A is a key mediator of this disassembly (Pugacheva et al., 2007). Despite the importance of these findings, several mechanistic details remain poorly defined, and our understanding of cilium disassembly has notably lagged behind the increasingly detailed picture of cilium assembly that has emerged (Zhao et al., 2023). Moreover, much remains unknown about the many other contexts in which cilia disassembly occurs.

Cilia disassembly and ciliogenesis have traditionally been studied in isolation, likely because they produce mutually exclusive outcomes, yet they share regulatory steps and act on common molecular components. Understanding either process therefore necessitates an integrated view of how their regulatory networks interconnect.

Impaired cilia formation or function causes ciliopathies – syndromic disorders with wide-ranging symptoms including patterning defects, multi-organ disruptions, metabolic imbalances and sensory and cognitive deficits (Braun and Hildebrandt, 2017; Reiter and Leroux, 2017). Disassembly defects, however, have not been clearly linked to canonical ciliopathies (Ott and Mukhopadhyay, 2025). Instead, recent evidence implicates excessive or insufficient disassembly in distinct pathological states, including cancers, neurological disorders and developmental defects, raising the possibility that modulating disassembly pathways could inform therapeutic strategies.

In this Review, we discuss recent insights into when and where cilia disassemble, delineate the molecular mechanisms that mediate cilia loss, and discuss the coordinated regulatory strategies that enable switching between ciliated and non-ciliated states. We focus on mammalian cilia disassembly but highlight some pertinent findings from other species. Finally, we discuss the roles of disassembly in disease and argue that emerging evidence for distinct pathological states driven by aberrant cilia disassembly supports creation of a new ciliopathy subclass: ‘disassembly-related ciliopathies.’

## When and where cilia disassemble

### Pre-mitotic and pre-meiotic disassembly

Although cilia can be long-lived organelles, they typically do not pass from a mother to a daughter cell. In cultured mammalian cells, developing tissues and unicellular organisms, such as *Chlamydomonas* (Box 1), cilia and flagella disassemble prior to mitosis (Bangs et al., 2015; Bloodgood, 1974; Kasahara and Inagaki, 2021; Long et al., 2025; Pan, 2023; Sanchez and Dynlacht, 2016). Early studies of quiescent cultured cells stimulated with serum mitogens used immunofluorecent staining to assess cilia disassembly as cells re-entered the cell cycle. Under these conditions, disassembly occurred either within ~2 hours of serum addition (at the G1/S transition) or ~12-24 hours later (in S/G2) (Fig. 1B) (Pugacheva et al., 2007; Spalluto et al., 2013; Tucker et al., 1979a; Tucker et al., 1979b). In one study, the frequency of ciliated cells rebounded partially after the first disassembly phase and then dropped again at the second, indicating that cilia regrowth can occur in S phase (Tucker et al., 1979b). More recent work examining cycling immortalized cells, organoids and developing tissues revealed cilia disassembly primarily in G2, prior to mitosis (Fig. 1B) (Ford et al., 2018; Ho et al., 2020; Ott et al., 2024a). Thus, although cilia loss is consistently observed before mitosis, its timing during the cell cycle can vary (Ford et al., 2018).

Cilia disassembly during meiosis has also been reported. In oocytes, primary cilia disassemble prior to or during the pachytene stage (Mytlis et al., 2022). In mouse spermatogenesis, primary cilia are found only on zygotene spermatocytes in the adult mouse but persist into the diplotene stage in prepubertal males (Mytlis et al., 2022; Pérez-Moreno et al., 2025). Notably, meiosis is characterized not only by cilia disassembly but also by centriole elimination or inactivation, especially in oogenesis (Avidor-Reiss et al., 2015).

What is the functional significance of cilia removal prior to mitosis and meiosis? The importance of centrioles in mitosis has led many to suggest that cilia disassembly might facilitate centriolar migration and bipolar spindle formation (Kasahara and Inagaki, 2021; Kim et al., 2015; Plotnikova et al., 2009). However, centrioles are not strictly required for mitosis in vertebrate cells and are absent in oocyte meiotic spindles (Bazzi and Anderson, 2014; Kalbfuss and Gonczy, 2023; Wong et al., 2015). Moreover, the axoneme can remain associated with the mother centriole in early mitosis of PtK1 (kangaroo rat) cells (Rieder et al., 1979), and cilia and flagella persist throughout mitosis in Trypanosomes and *Giardia intestinalis* (Dawson and House, 2010; Langousis and Hill, 2014). Pre-mitotic cilia disassembly might reset ciliary signaling or coordinate cilia regrowth in daughter cells. However, cilia-dependent signaling can persist through mitosis (Ho et al., 2020), and cilia reform asynchronously after mitotic exit, growing first from the centriole that templated the parental cilium (Anderson and Stearns, 2009; Paridaen et al., 2013). Thus, although cell cycle regulators promote cilia disassembly (as detailed below), the functional significance underlying this coordination merits further investigation.

### Differentiation-associated disassembly

Cilia are key signaling hubs (Hilgendorf et al., 2024; Mill et al., 2023), and many non-ciliated differentiated cells arise from ciliated progenitors (Ott and Mukhopadhyay (2025). Thus, loss of primary cilia during differentiation can fundamentally alter the signaling responsiveness of differentiated cells. The absence of cilia in differentiated cells can arise either through pre-mitotic disassembly coupled to suppression of ciliogenesis in daughter cells or through cell cycle-independent (post-mitotic) disassembly (Fig. 1C). Evidence for the latter comes from cerebellar granule cell (GC) progenitors, which lose cilia as they differentiate into neurons (Chang et al., 2019; Constable et al., 2024; Ott et al., 2024a). In this case, cilia loss serves to extinguish the mitogenic effects of ciliary Sonic Hedgehog (SHH) signaling (Shimada et al., 2018; Spassky et al., 2008). Importantly, the mechanisms governing post-mitotic disassembly appear to differ from those governing pre-mitotic disassembly, as discussed below.

### Stimulus-induced disassembly

Extrinsic signals, including the growth factors insulin-like growth factor 1 (IGF-1) and platelet-derived growth factor (PDGF), can stimulate cilia disassembly (Fig. 1A), in some cases without driving cell cycle progression (Pugacheva et al., 2007; Tucker et al., 1979b; Yeh et al., 2013). In epithelial cells, transforming growth factor beta (TGF-β) triggers cilia disassembly when combined with Ca^2+^ depletion, which causes an epithelial–myofibroblast transition (Rozycki et al., 2014). Activation of two G-protein coupled receptors (GPCRs) also induces robust cilia disassembly: the phospholipid lysophosphatidic acid (LPA), a key mitogenic component of serum, promotes disassembly through LPA receptor 1 (LPAR1) (Hu et al., 2021; Walia et al., 2019), while proteolytic activation of coagulation factor II receptor / protease activated receptor 1 (F2R/PAR1) likewise induces disassembly (Elliott et al., 2025). Both LPAR1 and F2R can trigger disassembly independent of the cell cycle, as disassembly proceeds even when S phase entry is blocked pharmacologically (Elliott et al., 2025). Interestingly, the F2R paralog F2RL1/PAR2 also promotes cilia disassembly, although by a distinct mechanism involving transcriptional downregulation of ciliary components, discussed below (Li et al., 2023).

External stresses and mechanical forces can also promote cilia disassembly. For example, in epithelial wound-healing assays, disassembly occurs in the cells adjacent to the wound that migrate to fill the gap (Grisanti et al., 2016). The length and frequency of tenocyte cilia decrease upon repeated extension and relaxation of the growth substrate (Rowson et al., 2018), and hyperosmotic shock induces cilia disassembly in cultured kidney cells (Otani et al., 2024). In these cases, cilia regrow when cells are returned to baseline conditions. Flow-induced cilia disassembly occurs in cultured human umbilical vein endothelial cells upon exposure to laminar shear stress (Iomini et al., 2004) and may also occur *in vivo* during heart valve development (Berg et al., 2025). In addition to physical stresses, infection can induce cilia disassembly, as cilia frequency decreases upon infection with porcine coronaviruses (Zhuang et al., 2025) or with the intracellular bacterium *Chlamydia trachomatis* (Ekka et al., 2024).

Stimulus-induced disassembly is not necessarily uniform. Disassembly frequency, kinetics and the extent of the response – shortening versus full disassembly – can all vary and depend in part on the cilia disassembly mechanisms that are engaged (Ho et al., 2023; Pugacheva et al., 2007; Tucker et al., 1979b). Additionally, many single-celled organisms also disassemble cilia via diverse mechanisms in response to cell stresses, developmental transitions and cell cycle progression (Box 1). In the next section, we discuss the mechanisms that mediate cilia disassembly before examining how cells balance cilia growth and disassembly to control ciliation state.

## Mechanisms of cilia disassembly

No single, linear disassembly pathway is used by all cells. Rather, cell state and disassembly stimuli likely determine which disassembly factors are activated and the fate of ciliary components. For example, whether the axoneme is depolymerized, severed or internalized varies, and the ciliary membrane can be either shed or internalized (Bloodgood, 1974; Mirvis et al., 2019; Rieder et al., 1979). In mammalian cells, detailed live-cell imaging of mitogen-induced disassembly revealed that multiple disassembly mechanisms can occur sequentially in individual cilia (Mirvis et al., 2019). Cilia disassembly further involves changes in the composition and structure of the basal body that promote disassembly and repress regrowth. The following sections examine these sub-structures separately, then discuss how changes to the ciliary microtubules and membrane are coordinated with events at the centrosome and cell body.

### Axoneme disassembly

**Figure 2:**
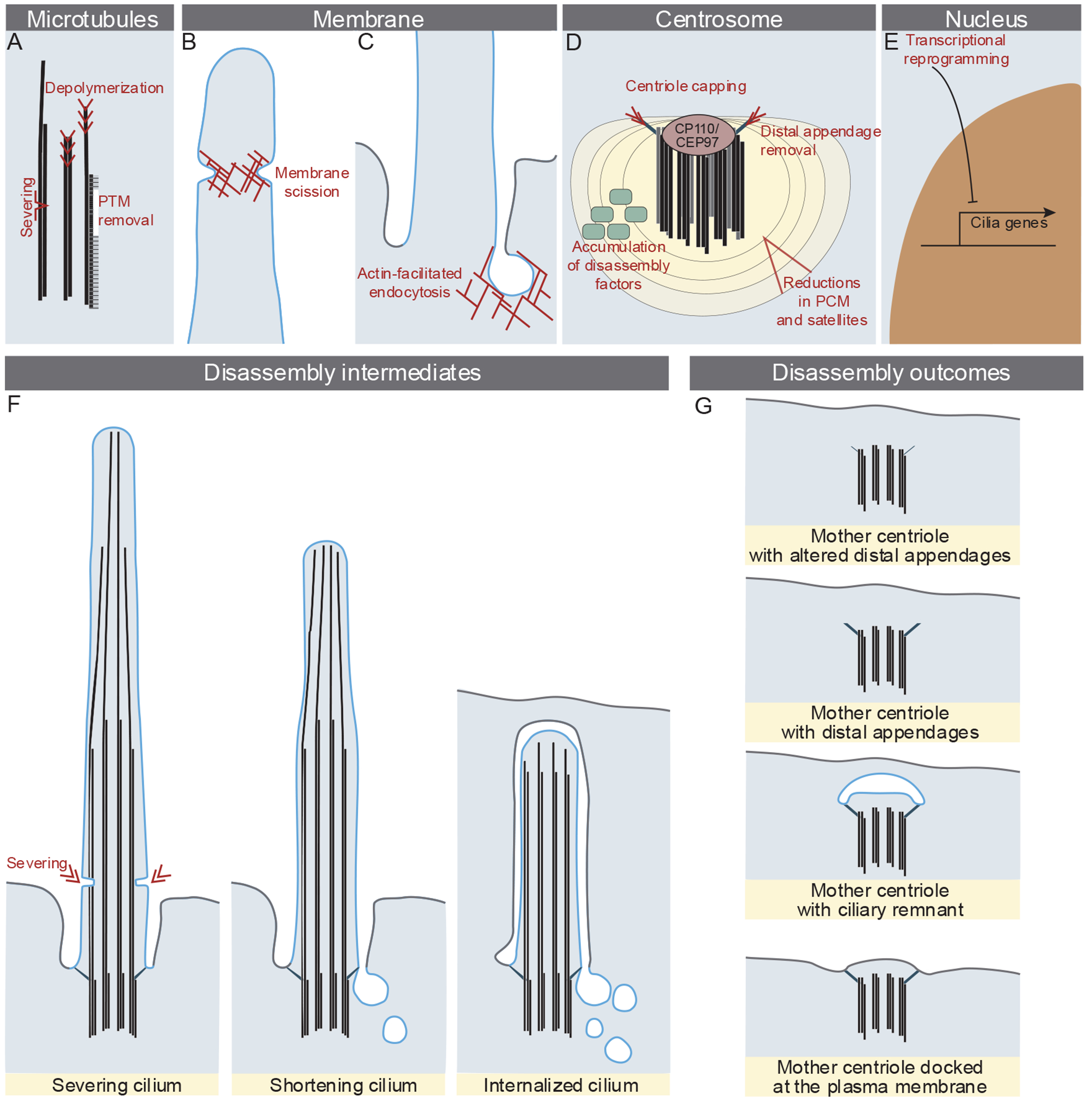
Mechanisms contributing to cilia disassembly (A) Axoneme disassembly can involve microtubule severing, depolymerization and/or removal of PTMs. (B) Branched actin polymerization promotes membrane scission events, which can remove just the cilium tip, as depicted here, or larger portions of the cilium, as in (F). (C) Ciliary membrane internalization during disassembly involves actin-facilitated endocytosis at the cilium base. (D) Centrosomal remodeling during disassembly can involve distal appendage removal, centriole capping by proteins such as CP110 and CEP97, and reduction of PCM and centriolar satellites. Disassembly factors can also accumulate at the basal body. (E) Transcriptional reprogramming can contribute to disassembly and/or prevent cilia regrowth via suppression of genes needed for cilia assembly or maintenance. (F) Because multiple mechanisms mediate cilia disassembly, different intermediate states are observed, including cilia undergoing severing (left), shortening (center), or internalization (right). (G) The final state of the mother centriole can vary in terms of its distal appendage composition and membrane association.

As the structural core of the cilium, the axonemal microtubules are a primary target of disassembly mediators. Several enzymes that depolymerize or sever microtubules, together with factors that alter axonemal PTMs, contribute to axoneme disassembly (Fig. 2A) (Mirvis et al., 2018; Zhang et al., 2025). Specifically, the depolymerizing kinesins KIF24 and KIF2A (and their orthologs) promote disassembly across multiple models and species (Box 1) (Ding et al., 2019; Kim et al., 2015; Miyamoto et al., 2015; Pan, 2023; Zhang et al., 2019). Overexpression or acute recruitment of the microtubule-severing enzymes spastin and katanin can also induce cilia disassembly (Liu et al., 2022; Mirvis et al., 2019). Spastin inactivation leads to longer and more frequent cilia in neurons and impairs resorption of motile cilia in the choroid plexus (Ho et al., 2023; Jeong et al., 2019). Further studies will be required to determine whether microtubule-severing enzymes are generally required for disassembly.

Although clearly implicated in disassembly, how axoneme severing or depolymerization is achieved mechanistically remains unclear. In many mammalian primary cilia, doublet microtubules in the proximal axoneme transition to singlets, with some terminating before they reach the tip (Kiesel et al., 2020; Ott et al., 2024b; Sun et al., 2019). Severing or depolymerizing singlet and doublet microtubules could have distinct requirements. Additionally, because axonemal microtubules extend from centriolar microtubules, their depolymerization must initiate from microtubule plus ends inside the cilium; however, KIF24 and KIF2A are enriched at the basal body. It remains unclear whether depolymerizing kinesins enter cilia to act at plus ends, suppress axoneme regrowth from the mother centriole, or remodel cytoplasmic microtubules to restrict cargo delivery to the basal body (Ding et al., 2019; Kim et al., 2015; Miyamoto et al., 2015; Zhang et al., 2019).

PTMs, including acetylation and glutamylation, influence the structure and physical properties of microtubules (de Jager et al., 2025; Wloga et al., 2017; Xu et al., 2017). Accordingly, enzymes that write or erase PTMs influence axoneme disassembly (Janke and Magiera, 2020; Wloga et al., 2017). Most notably, histone deacetylase 6 (HDAC6), which deacetylates α-tubulin, promotes cilia disassembly, and inhibition of HDAC6 or the deacetylase SIRT2 impairs disassembly (Pugacheva et al., 2007; Ran et al., 2015; Wloga et al., 2017). However, loss of HDAC6 or the acetyltransferase ATAT1 does not grossly alter ciliation *in vivo* (Bertrand and Grimes, 2026; Fukada et al., 2012; Kalebic et al., 2013; Kim et al., 2013; Lysyganicz et al., 2021; Zhang et al., 2008). Although axoneme deacetylation has been observed during flagellar disassembly in *Chlamydomonas*, it has not been documented during mammalian cilia resorption (L’Hernault and Rosenbaum, 1985). HDAC6 could also contribute to disassembly by deacetylating other proteins (Bershteyn et al., 2010; Kalebic et al., 2013; Ran et al., 2015; Zhang et al., 2008).

Reduced activity of tubulin glutamylases also destabilizes axonemes during pre-mitotic disassembly (He et al., 2025). However, acute deglutamylation does not trigger immediate cilium loss, and thus glutamylation removal is not sufficient to drive disassembly (Hong et al., 2018). Further studies are needed to determine whether changes in PTMs during disassembly directly destabilize the axoneme or promote disassembly by other means, such as tuning the binding and activity of microtubule-severing enzymes and depolymerizing kinesins. For example, glutamatylation has stoichiometry-dependent effects on spastin activity, illustrating the potentially complex interplay among these factors (Valenstein and Roll-Mecak, 2016).

In addition to active depolymerization of microtubules, work in *Chlamydomonas* indicates that baseline disassembly activity must be continuously counterbalanced to maintain flagellar length (Box 1). This balance depends on intraflagellar transport (IFT), a microtubule-motor-driven cargo transport system within cilia and flagella (Kozminski et al., 1993; Marshall and Rosenbaum, 2001). A similar balance may also be needed in mammalian cells, as acute inhibition of IFT using engineered, chemically inhibitable kinesin-II motors lead to progressive primary cilia loss (Engelke et al., 2019). In summary, multiple enzymes and mechanisms collaborate to promote axoneme disassembly, but key details remain to be elucidated.

### Ciliary membrane remodeling and scission

During disassembly, the ciliary membrane can be released by scission or internalized via resorption (Fig. 2B, C). Live-cell imaging has demonstrated scission of ciliary tips followed by ciliary resorption as well as whole-cilium shedding prior to mitosis (Das and Storey, 2014; Mirvis et al., 2019; Phua et al., 2017). In *Chlamydomonas*, ESCRT-mediated release of extracellular vesicles from the flagellum increases during resorption (Long et al., 2016), indicating that well-studied membrane remodeling machineries might be repurposed for cilia disassembly (Pan, 2023).

Mechanistically, the phosphoinositide composition of the ciliary membrane coordinates with actin polymerization to regulate membrane scission (Mirvis et al., 2018). The phosphoinositide 5-phosphatase INPP5E, a cilium-localized enzyme that converts phosphatidylinositol 4,5-bisphosphate (PI(4,5)P_2_) to phosphatidylinositol 4-phosphate (PI(4)P), inhibits membrane scission (Phua et al., 2017). PI(4,5)P_2_ and the small GTPase Rab7 accumulate in the cilium when ciliary INPP5E is reduced. Rab7 then facilitates F-actin accumulation and ciliary membrane severing (Wang et al., 2019a). As noted below, endocytic events at the base of the cilium also contribute to cilia disassembly.

### Ciliary disassembly from the basal body

As the structural foundation of the cilium and the access point for protein trafficking to and from the cilium, the basal body plays important roles in cilia disassembly (Fig. 2D). Several disassembly mediators noted above are recruited to the basal body during cilia disassembly. For example, KIF2A is recruited via a mitogenic signaling pathway in which class I phosphoinositide 3-kinase alpha (PI3Kα) produces phosphatidylinositol (3,4,5)-trisphosphate (PIP_3_) near the centrosome, thereby recruiting 3-phosphoinositide-dependent protein kinase-1 (PDK1) and activating protein kinase C iota (PKCι) (Conduit et al., 2024). PDK1 and PKCι then phosphorylate the centrosomal protein CEP170, which recruits KIF2A to the basal body (Conduit et al., 2024; Ding et al., 2019; Zhang et al., 2019).

During pre-mitotic disassembly, Aurora A also accumulates at the basal body, where it serves as a central mediator of disassembly. Recruitment of Aurora A to the basal body is enabled by a proposed “cilium disassembly complex” containing the centrosomal proteins CPAP/CENPJ, OFD1 and NDE1 (Gabriel et al., 2016). NDE1 and its paralog NDEL1 localize to centrosomes and promote cilia disassembly, perhaps through their established roles in regulation of cytoplasmic dynein (Doobin et al., 2016; Inaba et al., 2016; Kim et al., 2011). Activation of Aurora A is also promoted by trichoplein (TCHP) and peri-centrosomal Ca^2+^ signaling (Inoko et al., 2012; Plotnikova et al., 2012). The centrosome is thus a key site where upstream cues converge to mediate cilia disassembly.

The basal body is not only a recruitment hub for disassembly mediators but also is modified during disassembly. The kinases never-in-mitosis A-related kinase 2 (NEK2) and polo-like kinase 1 (PLK1), which accumulate at the mother centriole during G2, mediate displacement of distal appendage proteins such as CEP164 during pre-mitotic cilia disassembly (Bowler et al., 2019; Viol et al., 2020). Because distal appendage proteins promote ciliogenesis by enabling membrane docking and recruitment of the ciliogenesis mediator TTBK2, their removal represses cilia reassembly (Graser et al., 2007; Lo et al., 2019; Tanos et al., 2013; Yang et al., 2018). Loss of NEK2 causes incomplete disassembly and aberrant retention through mitosis of a ciliary remnant (a small, intracellular ciliary structure that remains anchored to the mother centriole), whereas NEK2 overexpression accelerates disassembly (Viol et al., 2020). Interestingly, the extent of distal appendage protein displacement varies across cell types, which might account for differences in ciliary remnant retention during mitosis (Paridaen et al., 2013; Viol et al., 2020). In cerebellar GC neurons, distal appendages are retained throughout cilia disassembly, and the mother centrioles remain docked at the plasma membrane. In these cells, capping of the distal end of the mother centriole by CP110/CEP97 is a terminal step that prevents axoneme growth, thereby providing an alternative means to prevent ciliogenesis (Constable et al., 2024; Ott et al., 2024a).

The pericentriolar material (PCM) and centriolar satellites surrounding the centrioles support both ciliogenesis and cilia maintenance, in part by serving as hubs for recruitment of centriolar and ciliary proteins (Begar et al., 2025; Prosser and Pelletier, 2020; Werner et al., 2017; Woodruff et al., 2014). Reduction of the PCM and elimination of centriolar satellites occur during post-mitotic cilium disassembly in differentiating GCs, coincident with loss of IFT components at the basal body (Fig. 2D) (Constable et al., 2024). This remodeling could reduce delivery of nascent proteins to the cilium, preventing cilia maintenance and promoting disassembly.

### Regulation of cilia disassembly from within the cell body

Although the cilium and basal body are the principal locations of disassembly, important contributions also occur in the cell body. In particular, intracellular signaling events link disassembly stimuli to disassembly signaling effectors. For example, cell surface receptors F2R and LPAR trigger cytoplasmic signaling cascades, leading to activation of the mitochondrial enzyme SARM1 and Ryanodine Receptor-mediated Ca^2+^ release from the endoplasmic reticulum (Elliott et al., 2025). Elevated cytoplasmic Ca^2+^ and calmodulin can activate Aurora A by enhancing its association with the scaffolding protein NEDD9/HEF1 (Plotnikova et al., 2012). In pre-mitotic disassembly, many mediators are directly linked to cytoplasmic signaling associated with mitogens and the cell cycle. For example, NEK2 and Aurora A have key roles in cell cycle progression in addition to cilia disassembly (Faragher and Fry, 2003; Nikonova et al., 2013). Similarly, CDK6 was recently shown to promote disassembly by limiting axoneme polyglutamylation, and PI3Kα promotes growth factor signaling as well as KIF2A activity (He et al., 2025).

Cytoskeletal alterations in the cell body also promote cilia disassembly (Mirvis et al., 2018; Smith et al., 2020). For example, cytoplasmic actin stimulates endocytic events at the cilium base that promote pre-mitotic disassembly (Fig. 2C). Specifically, phosphorylation of the dynein light chain Tctex-1upon S-phase entry promotes Arp2/3-mediated branched actin assembly for endocytosis at the ciliary pocket. In this pathway, Tctex-1 and activated CDC42 GTPase recruit Annexin A2 to locally activate Arp2/3 (Saito et al., 2017). Arp2/3 can also be recruited to the base of the cilium by deacetylated cortactin, an HDAC6 target (Ran et al., 2015). Additionally, F-actin can suppress assembly, possibly by inhibiting vesicular trafficking to the base of the cilium (Kim et al., 2010).

Moving from the cytoplasm to the nucleus, transcriptional reprogramming can mediate disassembly by downregulating expression of genes needed for cilia maintenance (Fig. 2E). This mechanism, termed “withdrawal of maintenance,” is exemplified by developmental cilia loss in the cerebellum (Constable et al., 2024). In differentiating GC neurons, reductions in IFT, PCM and centriolar satellite components coincide with and are at least partly driven by transcriptional downregulation of their encoding genes (Constable et al., 2024). Consistent with this model, expression of ciliary genes was recently shown to be a critical determinant of ciliation in the early mouse embryo (Liang et al., 2025).

Although cilia are typically maintained in multiciliated cells, transcriptional regulation of motile cilia disassembly has been reported. In the *Xenopus laevis* epidermis, NOTCH signalling-mediated transdifferentiation leads to repression of the key cilia-regulatory genes *foxj1* and *pcm1* (Tasca et al., 2021). In the mouse choroid plexus, cilia length and frequency decrease progressively from early postnatal stages through adulthood (Ho et al., 2023). Here, enzymes controlling axonemal PTMs are targeted: ATAT1 and tubulin glutamylases and glycylases are downregulated, whereas SIRT2 and the deglutamylase AGBL4 are upregulated. Also in the choroid plexus, mast cell-derived tryptase activates the GPCR F2RL1 to promote disassembly via suppression of the ciliary transcription factor FOXJ1 (Li et al., 2023). Given recent findings that the SP5 and SP8 transcription factors and the SWI/SNF chromatin remodeling complex promote ciliary gene expression and regulate ciliation (Cheng et al., 2025; Liang et al., 2025), how extensively transcriptional regulators contribute to cilia disassembly merits further investigation.

### Multi-faceted disassembly of cilia

Cilium disassembly requires coordinated changes in the axoneme, ciliary membrane, basal body and cell body. How these events are integrated remains unclear, partly because the mechanisms, order of events and intermediate states vary across physiologic contexts (Fig. 2F). Nonetheless, recent studies have begun to reveal mechanisms that link some of the disassembly processes described above. For example, NEK2 promotes both axoneme disassembly via KIF24 and distal appendage removal, thereby coupling these processes (Kim et al., 2015; Viol et al., 2020). This example illustrates coordinated activation of disassembly and inhibition of assembly (further discussed below). Initial events in disassembly might also facilitate downstream steps. For example, scission of the ciliary membrane might enable axoneme disassembly by removing tip structures that stabilize axonemal microtubules (Saunders et al., 2025).

Ciliogenesis pathways and ciliary structures vary by cell type; similarly, disassembly mechanisms likewise can be specified by cell type and lead to different endpoints (Fig. 2G). Disassembly often results in a free cytoplasmic mother centriole; however, neural progenitors retain a ciliary remnant after disassembly, and PtK1 cells exhibit internalized membrane-less axonemes during disassembly (Paridaen et al., 2013; Rieder et al., 1979). In GCs, many cilia are internalized (concealed from the cell surface) in the late stages of disassembly, and the mother centriole ultimately docks at the cell surface (Ott et al., 2024a). These contextual differences are not only important mechanistically but also determine the fate of ciliary components – e.g. whether membrane-embedded receptors and axonemal building blocks are shed or resorbed into the cell and potentially recycled (Ott et al., 2024a; Rieder et al., 1979).

Upstream signals might be crucial mechanistic determinants, dictating which disassembly factors become active. For example, cell cycle kinases are not active in post-mitotic cells, so alternatives to Aurora A-based signaling are needed to drive disassembly. Consistent with this model, GC progenitors employ a pre-mitotic disassembly program during proliferation and then switch to a withdrawal-of-maintenance mode during post-mitotic differentiatiation (Constable et al., 2024). In GPCR-initiated disassembly, the paralogous receptors F2R and F2RL1 induce different disassembly mechanisms (Elliott et al., 2025; Li et al., 2023). A key challenge is thus to determine how information is relayed from disassembly stimuli sensors to disassembly mediators and how cell type and state impact this process.

## Integrating cilium assembly and disassembly signaling to control ciliation

Cilia disassembly switches cells from a ciliated to a non-ciliated state, whereas ciliogenesis brings about the opposite transition. A useful way to conceptualize this system is as a bistable switch, in which both states are stable and transitions require signals that exceed a cell state-dependent threshold. Typically, stimuli that alter ciliation jointly modulate both assembly and disassembly pathways: processes that promote disassembly counteract those that promote ciliogenesis and vice versa. In this section, we examine the mechanisms and consequences of this mutual antagonism between assembly and disassembly pathways. We also discuss factors that influence a cell’s ability to switch between ciliated and non-ciliated states to generate transient or persistent changes in ciliation.

### Mutual antagonism of cilium assembly and disassembly

**Figure 3:**
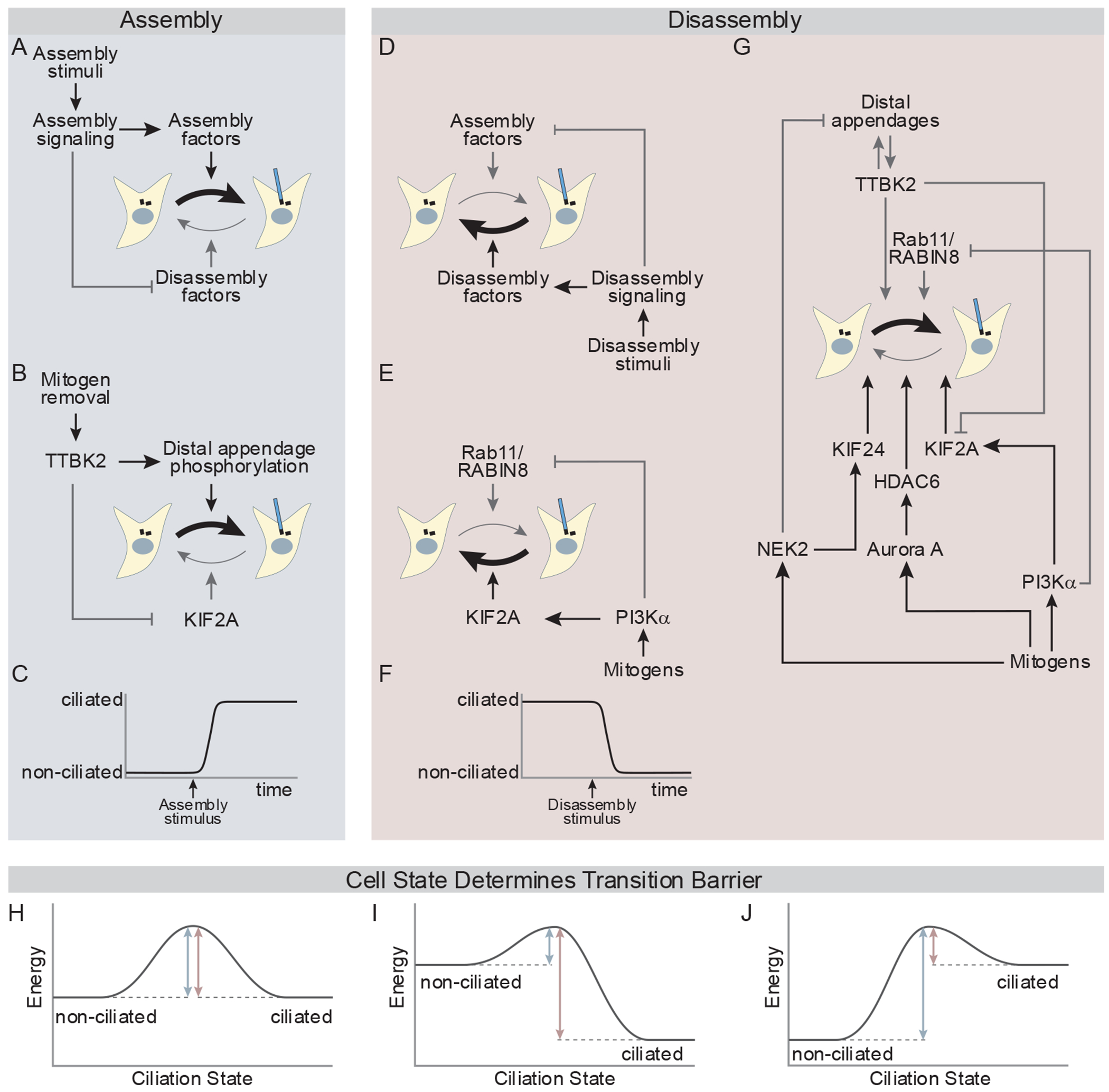
Assembly and disassembly signaling regulate switching between ciliated and non-ciliated states (A-C) Assembly signaling pathways drive transition from a non-ciliated to a ciliated state (C) by activating assembly factors and inhibiting disassembly factors (A). For example, mitogen removal activates TTBK2, which phosphorylates both distal appendage proteins to promote ciliogenesis and KIF2A to inhibit microtubule depolymerization (B). (D-F) Disassembly signaling pathways drive transition from a ciliated to a non-ciliated state (F) by inhibiting assembly factors and activating disassembly factors (D). For example, mitogens activate PI3Kα, recruiting KIF2A to the basal body for disassembly and blocking pro-assembly Rab11-RABIN8 association (E). (G) A single disassembly stimulus can activate multiple signaling modules in parallel. As illustrated here, mitogens activate Aurora A, NEK2 and PI3Kα. Aurora A activates HDAC6, while NEK2 enhances KIF24-dependent disassembly and promotes distal appendage remodeling, leading to loss of ciliogenesis-promoting TTBK2 activities (as in B). In parallel, PI3Kα (also shown in E) promotes KIF2A activity and prevents Rab11-RABIN8 association. (H-J) When ciliation state is modeled as a bistable system, the barrier to switching between ciliated and non-ciliated states can be represented as the height of an energy barrier. In (H), both states are equally favored, so similar levels of assembly or disassembly stimulus are required to change state. Cellular processes such as metabolism, transcriptional regulation and cell cycle progression can shift the relative favorability of the two states and thereby alter transition barriers, as shown in (I) and (J). In such cases, less stimulus is needed to switch into the more favored state than into the less favored state.

Ciliogenic signaling pathways frequently inhibit disassembly factors, creating a coherent feed-forward module in which parallel pathway branches promote a common outcome (Fig. 3A) (Alon, 2007). For example, mitogen removal activates TTBK2, which phosphorylates the Rab8-activating guanine nucleotide exchange factor RABIN8 and distal appendage proteins (Bernatik et al., 2020; Lo et al., 2019). This pathway might thereby stimulate ciliogenesis by promoting vesicle recruitment to the mother centriole (Cajanek and Nigg, 2014; Goetz et al., 2012). Active TTBK2 also phosphorylates KIF2A, reducing its depolymerizing activity and ability to promote disassembly (Fig. 3B) (Benk Vyslouzil et al., 2025; Watanabe et al., 2015). Mitogen removal additionally promotes degradation and/or relocation of disassembly factors such as NDEL1 and TCHP, thus restraining Aurora A-mediated disassembly (Inaba et al., 2016; Kasahara et al., 2014). Cilia stability is further supported as ciliogenesis proceeds by accumulation of axonemal PTMs, decreasing the probability of disassembly (Gadadhar et al., 2017; He et al., 2018). We propose that ciliogenic signaling pathways collectively shift a cell from a non-ciliated to a ciliated state in a switch-like manner by coordinating activation of assembly factors and suppression of disassembly factors (Fig. 3C).

Conversely, disassembly signaling can concurrently activate disassembly factors and repress assembly factors (Fig. 3D). For example, mitogen-activated PI3Kα signaling promotes disassembly via CEP170 and KIF2A (Conduit et al., 2024; Walia et al., 2019) and simultaneously diverts the small GTPase Rab11 from the ciliogenic effector RABIN8 to the non-ciliogenic effector WDR44 (Fig. 3E) (Accogli et al., 2024; Walia et al., 2019). These parallel branches cooperate to favor the non-ciliated state prior to mitotic entry. Multiple mechanisms similarly act together to promote cilia loss during differentiation. For example, cilia loss in differentiating cerebellar GC neurons is characterized by CEP97-mediated centriole capping as well as transcriptional down-regulation of ciliogenic factors (Constable et al., 2024). Thus, disassembly stimuli often simultaneously activate disassembly and repress assembly pathways, thereby switching cells from ciliated to non-ciliated states (Fig. 3F). Furthermore, a stimulus can lead to the activation of several downstream pathways. Fig. 3G illustrates multiple mitogen-activated disassembly modules that function in parallel to reinforce disassembly while inhibiting assembly. In addition to stimulating KIF2A recruitment through PI3Kα (Conduit et al., 2024) (Fig. 3E), mitogens also activate Aurora A and NEK2, which target HDAC6 and KIF24, respectively (Kim et al., 2015; Pugacheva et al., 2007). PI3Kα and NEK2 not only promote disassembly but also repress assembly via inhibition of RABIN8 and displacement of distal appendage proteins (Fig. 3G) (Bowler et al., 2019; Viol et al., 2020; Walia et al., 2019). Extensive branching within these signaling networks could thus promote robust and persistent responses to pathway activation.

### Modeling regulation of ciliation state as a bistable landscape

Because ciliogenesis and cilia disassembly drive transitions between ciliated and non-ciliated cellular states, we hypothesize that the mutually antagonistic feed-forward modules governing assembly and disassembly could generate a bistable system. In this model, the presence or absence of a cilium is maintained until a stimulus produces sufficient assembly or disassembly activity to switch states. The activation threshold for such switching can be conceptualized as the height of an energy-like barrier separating non-ciliated and ciliated states, with the depth of each well reflecting the relative stability of that state (Fig. 3H). Fig. 3I and 3J illustrate hypothetical landscapes in which the ‘favorability’ or ‘stability’ of ciliated versus non-ciliated states differs, such that transitions in one direction encounter a smaller barrier than the reverse transition. In such landscapes, modest inputs can bias cells toward the more favorable state, whereas larger inputs are needed to drive the system in the other direction.

Within this framework, both the magnitude of external stimuli and the landscape set by the internal cellular state are tunable features. Gene expression programs can thus strongly influence the baseline landscape, such that stimuli that strongly promote ciliation in some cell types can have no effect in other cell types (Bangs et al., 2015). In the early mouse embryo and the retina, lineage-restricted expression of assembly genes underlies cell type-specific ciliation (Liang et al., 2025; Liu et al., 2025; Zhang et al., 2026). Gene expression changes can also drive long-term shifts in ciliation, exemplified by permanent cilia loss during cerebellar GC neuron differentiation (Constable et al., 2024).

Other features of the internal cellular state, such as metabolic status, cell cycle stage and activated signaling pathways, can bias the system toward either the ciliated or non-ciliated state by altering transition thresholds or state stability. Mitogenic signaling provides a sufficiently strong stimulus to drive disassembly even in highly ciliated lineages. Cell cycle-linked feed-forward and positive-feedback motifs might ensure that, once triggered, disassembly persists through mitosis (Tyson and Novak, 2001). Upon mitotic exit, cell cycle factors are inactivated or degraded along with the components sustaining disassembly, allowing cilia re-formation (Kasahara and Inagaki, 2021). Similarly, distal appendage remodeling during pre-mitotic disassembly might ensure that ciliogenesis is suppressed until distal appendages are restored after mitotic exit (Bowler et al., 2019; Viol et al., 2020).

Bistability offers a useful framework for understanding cilia dynamics, and assembly and disassembly pathways clearly antagonize one another. However, current data are insufficient to formally claim that the ciliation system is bistable. Future experiments should test whether positive feedback loops, cooperative or ultrasensitive signaling and/or mutual inhibitory motifs exist within this network, as these features are typically required to generate true bistability (Ferrell, 2002). An additional testable prediction of this type of switch is hysteresis, in which the transition threshold depends on the system’s history.

Evidence in support of mutual inhibition can be seen in cellular responses to perturbations. Genetic or chemical inhibition of disassembly increases ciliogenesis in proliferating cells and promotes cilium elongation in quiescent cells. Such phenotypes are seen upon inhibition of actin polymerization (Kim et al., 2010; Smith et al., 2020), SARM1 (Elliott et al., 2025), NEK2 (Kim et al., 2015), KIF24 (Kim et al., 2015), TCHP (Inoko et al., 2012; Yamakawa et al., 2021) or NDE1/NDEL1 (Doobin et al., 2016; Inaba et al., 2016). These observations suggest that disassembly-promoting factors have basal activity that restrains cilia growth, such that acute inhibition of disassembly unmasks an underlying capacity for cilia growth. Constitutive disassembly activity is also supported by observations of cilia loss following acute disruption of cilia assembly or maintenance – for example, by interrupting IFT or expressing dominant-negative Rab34 (Engelke et al., 2019; Ganga et al., 2021; Parker and Quarmby, 2003). Conversely, activation of disassembly can induce transient cilia loss, for example upon activation of F2R or treatment with PDGF (Elliott et al., 2025; Tucker et al., 1979b). Cilia reform upon termination of the disassembly stimuli, possibly due to relief of assembly inhibition. Together, these data suggest that assembly and disassembly function as mutual inhibitory motifs.

Notably, in several cases, suppressing cilia disassembly can partially rescue defects in cilia assembly or maintenance, consistent with expectations for a bistable system. For example, impaired ciliation in cells carrying a hypomorphic mutation in the IFT-B gene *Ift88* is partially rescued by the actin polymerization inhibitor cytochalasin D (Kim et al., 2010). Actin inhibition similarly rescues ciliary defects in cells lacking the IFT-A subunit WDR35 or expressing mutant Rab34 (Fu et al., 2016; Ganga et al., 2021). *IFT88, WDR35* and *RAB34* are ciliopathy genes (Bruel et al., 2023; Reiter and Leroux, 2017); thus, targeting disassembly pathways could have therapeutic potential.

#### Physiologic and pathologic roles of cilia disassembly

In this section, we highlight emerging evidence that both excessive and insufficient disassembly can drive disease (summarized in Table 1). Because perturbing disassembly is mechanistically distinct from disrupting ciliogenesis, disorders caused by dysregulated disassembly need not always resemble conventional ciliopathies. We also discuss how growing insight into disassembly pathways and their links to disease suggests new opportunities to therapeutically target disassembly-driven pathologies.

### Excessive cilia disassembly

Excessive cilia disassembly can reduce ciliation and alter ciliary signaling. Importantly, the impact of excessive disassembly likely differs from that of defective assembly and produces distinct disease states. For instance, excess disassembly might reduce overall ciliation but permit ciliary function within limited time windows or sub-populations of cells. Consequently, pathologies caused by excessive disassembly can present with clinically distinct symptoms and have not been consistently classified as canonical ciliopathies. The examples below illustrate a general principle: when factors that normally control disassembly are hyperactivated, cilia can become too rare or transient to sustain normal signaling, even if the core ciliogenesis machinery is intact.

Activating mutations of the PI3Kα gene *PIK3CA* in *PIK3CA*-related overgrowth syndrome (PROS) illustrate how excessive disassembly might contribute to disease (Conduit et al., 2024). In PROS, some clinical features resemble those seen in canonical ciliopathies, such as polydactyly. This similarity is consistent with the aberrant loss of cilia observed in a murine model of PROS, in which hyper-active PI3Kα promotes disassembly through PKCι-mediated phosphorylation of CEP170 and KIF2A (Fig. 3E). Here, decreased ciliation also impairs SHH signaling and neural tube closure, which could contribute to PROS symptoms (Conduit et al., 2024). Another branch of PI3Kα signaling inhibits cilia assembly by inducing Rab11 association with WDR44 instead of the ciliogenic effector Rabin8 (Fig. 3E) (Walia et al., 2019). Altered WDR44 activity might therefore contribute to cilia loss in PROS, a possibility supported by the recent finding that *WDR44* gain-of-function mutations cause a syndromic disorder via impaired ciliogenesis (Accogli et al., 2024).

Recently, activating mutations underlying the neurological disorder focal cortical dysplasia (FCD) were found to drive excess disassembly (Elliott et al., 2025). In FCD, somatic activating mutations disrupt cortical development and function, resulting in local brain lesions that induce seizures (Iffland and Crino, 2017). In a cohort of individuals with FCD (Chung et al., 2023), somatic mutations were found in genes that encode components of the F2R-initiated cilia disassembly pathway, including SARM1, RHOA, RYR2 and RYR3 (Elliott et al., 2025). FCD-linked variants in SARM1 and RHOA were further found to potentiate cilia loss. Furthermore, activating mutations in the mTORC1 pathway – a major cause of FCD – also cause cilia loss in FCD brain tissue (Di Nardo et al., 2020; Lim et al., 2015) and induce cilia loss in cultured cells via SARM1 (Elliott et al., 2025). Thus, excess cilia disassembly signaling is a recurring feature of FCD that might contribute to pathogenesis. Although further studies are needed, cilia loss might disrupt the important roles of cilia in neural progenitors, cortical development and regulation of neuronal excitability (Dyke et al., 2025; Hasenpusch-Theil and Theil, 2021; Liu et al., 2021; Tereshko et al., 2021; Wilsch-Brauninger and Huttner, 2021).

Aberrant stimulation of disassembly pathways by local signals has also been linked to pathology stemming from cilia loss. In tumor-associated hydrocephalus (TAH), an often lethal complication of brain metastasis, tumor-induced secretion of tryptase activates the receptor F2RL1 on choroid plexus epithelial cells, leading to inhibition of FOXJ1 (Li et al., 2023). Subsequent transcriptional down-regulation of ciliary components leads to disassembly, which in turn deregulates cerebrospinal fluid secretion, causing hydrocephalus.

Cilia loss in several cancers, including pancreatic ductal adenocarcinoma (PDAC) (Kobayashi et al., 2017; Seeley et al., 2009), breast cancer (Kim et al., 2015; Menzl et al., 2014), glioma (Goranci-Buzhala et al., 2021), melanoma (Zingg et al., 2018) and cholangiocarcinoma (Gradilone et al., 2013), has been linked to increased cilia disassembly and/or suppression of ciliogenesis (see Kiseleva et al. (2023) for further discussion of cilia in cancer). In breast cancer cells, increased NEK2 activity promotes cilia disassembly through KIF24 (as in Fig. 3G) (Kim et al., 2015). In melanoma, mutation of the methyltransferase EZH2 or increased expression of the chromatin regulator PRAME represses expression of cilia assembly and maintenance genes (Heo et al., 2026; Zingg et al., 2018). Notably, PI3Kα activating mutations found in PROS are also common in cancers, suggesting that these mutations might promote disassembly in diverse contexts (Samuels et al., 2004). In several instances, restoring cilia – for example, by inhibiting cilia disassembly factors – has been found to reduce tumor proliferation or promote differentiation, indicating that disassembly functionally contributes to tumorigenesis (Goranci-Buzhala et al., 2021; Kim et al., 2015; Zingg et al., 2018).

Preliminary evidence also suggests that inappropriate activation of disassembly pathways in post-mitotic neurons might contribute to neuronal vulnerability in degenerative diseases. Recently, mutations in *NEK1* and *CFAP410* associated with amyotrophic lateral sclerosis (ALS) were linked to cilia loss on motor neurons (De Decker et al., 2025; Noh et al., 2025). Although the precise ciliary functions of NEK1 and CFAP410 are not yet defined, mutant NEK1 might promote disassembly through Aurora A activation, as HDAC6 inhibitors restore cilia in cells with *NEK1* mutations (Noh et al., 2025). Increased disassembly (and reduced assembly) has also been observed in cells with Parkinson’s Disease-associated mutations in the kinase LRRK2, suggesting that dysregulated disassembly could also contribute to pathogenesis of this neurodegenerative disease (Sobu et al., 2021).

### Impaired cilia disassembly

Failure to properly disassemble cilia might also contribute to disease pathogenesis. Defective disassembly is frequently linked to microcephaly, in which impaired proliferation leads to reduced brain size. A microcephaly-linked mutation in CENPJ impairs cilia disassembly and delays G1-S cell cycle progression, causing premature differentiation of neural progenitors and diminished cortical development (Gabriel et al., 2016). Microcephaly-causing mutations also occur in NDE1 and WDR62. NDE1 is a dynein regulator that restricts cilia growth, and WDR62 promotes KIF2A recruitment to CEP170 at the basal body during disassembly (Kim et al., 2011; Zhang et al., 2019). Mutations in these genes increase ciliation, altering the proliferation and fate of neural progenitors (Doobin et al., 2016; Zhang et al., 2019). Lastly, *Lpar1* mutant mice display microcephaly, suggesting that LPA signaling regulates disassembly to facilitate progenitor expansion (Hu et al., 2021).

Insufficient disassembly during differentiation may also promote tumorigenesis. For example, although differentiated GC neurons are non-ciliated, tumor cells in the SHH class of medulloblastomas remain ciliated (Han et al., 2009). The mechanistic basis for aberrant ciliation is not clear, but a plausible model is that sustained SHH signaling drives activity of the transcription factor ATOH1 (Forget et al., 2014), which in turn promotes cilia maintenance by upregulating centriolar satellite function (Chang et al., 2019). Another non-ciliated cell type, adipocytes, also illustrates the consequences of impaired disassembly during differentiation. Normally, TCHP and Aurora A signaling promote disassembly during adipocyte differentiation; however, mutation of *Tchp* in mice causes alterations to pre-adipocyte ciliation, adipogenesis and metabolic responses to high-fat diet (Yamakawa et al., 2021). We anticipate identification of additional pathological consequences of dysregulated disassembly as our mechanistic understanding advances.

### Linking ciliary alterations to disease pathogenesis

Current efforts to classify ciliopathies distinguish between ciliopathies arising from mutations in proteins that localize primarily to cilia or centrosomes (first-order ciliopathies) and ciliopathies caused by mutations in non-ciliary proteins that facilitate ciliogenesis or cilia function (second-order ciliopathies) (Mill et al., 2023; Reiter and Leroux, 2017). An additional subclass – diseases with ciliary contribution – includes diseases in which ciliary dysfunction is part of a broader pathogenic mechanism (Lovera and Luders, 2021). Pathologies caused by cilia disassembly defects do not fit simply into these categories. Cilia- and centrosome-localized proteins as well as regulators elsewhere in the cell can alter disassembly and cause disease. In addition, pathogenic mutations can perturb multi-functional proteins like kinases, thus affecting both cilia disassembly and non-ciliary processes, similar to diseases with ciliary contributions. Furthermore, altered cilia disassembly may produce different clinical manifestations than those seen in canonical ciliopathies. We therefore propose creation of an additional ciliopathy subclass: disassembly-related ciliopathies.

As we learn more about cilia disassembly, we anticipate that additional diseases will be considered for this new category. A key challenge will be to determine whether changes in cilia frequency cause or accompany disease. For example, a variety of mutations in FCD-linked genes cause cilia disassembly, and decreased ciliation is observed in brain tissue of individuals with FCD. However, specific disease features that are directly attributable to cilia loss have not been definitively established (Di Nardo et al., 2020; Elliott et al., 2025; Lim et al., 2015). In particular, careful studies are needed to determine whether observed phenotypes arise from disrupted ciliary disassembly *per se* or from cilia-independent functions of the same molecular components. For example, *WDR62, PIK3CA* and *NEK1* regulate both ciliary and non-ciliary processes (Fry et al., 2012; Jayaraman et al., 2016; Morin et al., 2024). A compelling connection was established in the case of mTOR-driven FCD, in which knockdown of the ciliary regulator OFD1 restored cilia and rescued some cortical patterning defects (Lim et al., 2015). In melanoma, EZH2-driven cilia loss appears to have a causal role in tumorigenesis: EZH2 inhibition restores cilia and slows tumor growth, but these anti-tumor effects are abolished when ciliogenesis is disrupted (Zingg et al., 2018). To be considered a ciliopathy, the disruptions in disassembly regulation should contribute, at least in part, to disease pathogenesis. Functional assessment of causal roles will become increasingly feasible as tools to selectively modulate ciliation state are developed.

Another important challenge will be to determine the mechanisms by which changes in ciliation produce specific clinical features. In some cases, altered cell signaling might play a central role. For example, cilia loss can increase oncogenic WNT signaling in melanoma, whereas cilia retention may drive proliferative SHH signaling in medulloblastoma (Chang et al., 2019; Kiang et al., 2025; Ott et al., 2024a; Zingg et al., 2018). In other cases, including microcephaly, impaired disassembly reduces proliferative capacity and alters cell fate. Improved knowledge of normal ciliary signaling pathways will facilitate mapping of specific disassembly defects onto defined clinical outcomes.

### Therapeutic opportunities targeting cilia disassembly

Evidence that dysregulated disassembly can drive or contribute to disease indicates that targeting disassembly pathways has therapeutic potential. For excessive disassembly, pathway inhibition can protect cilia and reduce disease pathogenesis. In TAH, tryptase inhibitor treatment prevents PAR2 activation, preserves choroid plexus cilia and ameliorates hydrocephalus (Li et al., 2023). In glioma cells, inhibiting disassembly increases ciliation and promotes differentiation (Goranci-Buzhala et al., 2021). Ongoing efforts to develop small-molecule inhibitors of PI3Kα and SARM1 could similarly enable intervention for patients with PROS and FCD. Because assembly and disassembly signaling are interconnected, inhibiting disassembly may also offer therapeutic benefits when ciliogenesis is impaired. Consistent with this model, suppressing disassembly through inhibition of actin polymerization, Rho-associated protein kinases, or CDK6 can restore ciliogenesis in cells with mutation or depletion of the ciliopathy genes IFT88, RAB34, RPGRIP1L, or ARMC9 (Ganga et al., 2021; He et al., 2025; Kim et al., 2010; Smith et al., 2025).

Conversely, promoting disassembly could be advantageous in diseases with impaired disassembly or cilia-driven pathogenic signaling. Tumors dependent on cilia-mediated SHH signaling, such as medulloblastoma and basal cell carcinoma, might be slowed by therapeutics that trigger disassembly (Han et al., 2009; Wong et al., 2009). Similarly, disassembly-promoting therapies could benefit individuals with diseases involving aberrant ciliary signaling. In PKD, defects in the ciliary PKD1-PKD2 complex drive kidney cystogenesis (Boletta and Caplan, 2025; Clearman et al., 2023). In the cilia-dependent cyst activation model of PKD, loss of PKD1-PKD2 causes pathological ciliary signaling, and blocking ciliogenesis reduces cyst growth and disease progression (Ma et al., 2017; Ma et al., 2013). These effects suggest that pharmacologically enhancing disassembly might achieve similar benefits. Augmenting disassembly could also mitigate consequences of impaired disassembly in disorders such as microcephaly. More broadly, these considerations argue that context-dependent tuning of disassembly – either inhibition or promotion – could create valuable tools to treat diseases caused by dysregulated ciliation.

## Summary and outlook

The recent advances discussed in this Review have transformed the study of cilia disassembly by broadening our understanding of where, when and how cilia disassemble. Although important questions remain (Box 2), this expanded view of cilia disassembly is poised to drive advances in defining its underlying molecular mechanisms and in translating disassembly control into diagnostic and therapeutic opportunities.

## Figures and Tables

**Table 1.** Disassembly-related ciliopathies

Condition	Pathology	Key genes/proteins	Proposed mechanism
**Excess disassembly**			
PI3K-related overgrowth syndrome (PROS)	Malformations of adipose, lymphatic and vascular tissue; ventriculomegaly and polydactyly	PIK3CA	PIK3CA activating mutations promote Cep170 phosphorylation and KIF2A activation, thereby enhancing microtubule depolymerization at the basal body and increasing cilia disassembly.
Focal cortical dysplasia (FCD)	Focal lesions in the cerebral cortex caused by somatic mutations during development, leading to epileptic seizures	mTORC1 pathway, RHOA, SARM1, RYR2, RYR3	Somatic mutations aberrantly activate mTORC1 or other factors that promote cilia disassembly, driving excessive cilia disassembly and reducing ciliation of cortical progenitors.
Tumor-associated hydrocephalus	Hydrocephaly with increased intracranial pressure caused by brain metastasis of various tumors	F2RL1 (PAR2), Tryptase, FOXJ1	Tumor-induced tryptase activates F2RL1 and suppresses FOXJ1 target gene expression, leading to loss of choroid plexus cilia through increased disassembly and failed cilia maintenance.
Melanoma, pancreatic and breast cancers, glioma	Cancers of various tissues and origins	EZH2, NEK2, CENPJ, AURKA	Oncogenic alterations increase cilia disassembly and decrease ciliation, thereby permitting hyperactive proliferative and oncogenic signaling.
Amyotrophic lateral sclerosis (ALS)	Progressive neurodegenerative disease characterized by loss of motor neurons needed for muscle contraction	NEK1, CFAP410	Heterozygous variants in NEK1 and CFAP410 reduce ciliation, possibly by enhancing cilia disassembly.
**Insufficient disassembly**			
Microcephaly	Reduced brain size due to impaired neural differentiation, proliferation, and/or survival	WDR62, CENPJ, NDE1	Mutations in these centrosomal proteins impair cilia disassembly, leading to reduced proliferation and premature differentiation of neural progenitors.
SHH-driven medulloblastoma	Brain cancer arising from uncontrolled growth of cerebellar neural progenitors	SMO, PTCH1, SUFU, GLI2	Constitutive SHH pathway activation occurs in cells that often retain primary cilia, with insufficient disassembly promoting sustained mitogenic SHH signaling.

## Box 1: Pioneering work in disassembly of cilia and flagella from unicellular organisms

Cilia and flagella occur across diverse eukaryotic taxa and were likely present in the last eukaryotic common ancestor (Satir et al., 2008). Many insights into disassembly have come from unicellular systems, such as *Chlamydomonas reinhardtii* (Rosenbaum et al., 1969), *Giardia lamblia* (Dawson and House, 2010), *Naegleria gruberi* (Woglar et al., 2025) and chytrid fungi (Venard et al., 2020). In these organisms, disassembly can proceed through distinct routes, including gradual resorption, axonemal severing and retraction of the axoneme into the cytoplasm (Bloodgood, 1974; Long et al., 2025; Venard et al., 2020).

Disassembly mechanisms have been most extensively studied in the biflagellate green alga *Chlamydomonas*, which undergoes pre-mitotic resorption or stress-induced deflagellation by shedding (Pan, 2023; Quarmby and Mahjoub, 2023). Several elements of disassembly are evolutionarily conserved, and many insights in *Chlamydomonas* presaged similar findings in mammalian cells. For example, a key role for Ca^2+^ signaling was established in *Chlamydomonas* (Pan, 2023; Quarmby and Mahjoub, 2023). Furthermore, kinases homologous to Aurora A and NEK2 were identified as effectors for flagellar disassembly in *Chlamydomonas* before they were implicated in mammalian cilia disassembly (Mahjoub et al., 2004; Pan, 2023; Pan et al., 2004; Quarmby and Mahjoub, 2023; Wang et al., 2017). Changes in PTMs also contribute to disassembly in *Chlamydomonas*, including tubulin deacetylation, ubiquitination and methylation, although only ubiquitination has been explored in mammalian systems to date (Huang et al., 2009; L’Hernault and Rosenbaum, 1985; Mizuno and Sloboda, 2017; Schneider et al., 2008; Wang et al., 2019b).

IFT was also first linked to disassembly in *Chlamydomonas* (Dentler, 2005). IFT particles entering disassembling flagella lack cargo, restricting flagellar maintenance while promoting removal of flagellar components (Pan and Snell, 2005). IFT also promotes disassembly by mediating import of the microtubule depolymerase kinesin-13 (Piao et al., 2009) and disassembly-promoting kinases (Zhao et al., 2020). Because flagellar maintenance requires constant import, perturbing IFT promotes disassembly (Marshall and Rosenbaum, 2001). Inactivation of the *Chlamydomonas* KIF3A homolog causes deflagellation unless extracellular Ca^2+^ levels are low, in which case flagella gradually resorb (Parker and Quarmby, 2003). These experiments provided early evidence that cessation of ciliary maintenance triggers disassembly.

Reciprocal regulation of flagellar growth and shortening was also revealed by classic experiments in which one *Chlamydomonas* flagellum was severed. While the severed flagellum begins to regrow, the unsevered flagellum disassembles until it becomes shorter than the regenerating flagellum, after which both grow at similar rates (Coyne and Rosenbaum, 1970; Rosenbaum et al., 1969). This behavior inspired the “balance-point” model of flagellar growth, in which steady-state flagellar length is determined by length-independent shortening and length-dependent growth (Marshall and Rosenbaum, 2001). Importantly, these rates are not fixed: the kinases CNK2 (NEK family) and LF4 tune both assembly and disassembly in response to flagellar length perturbations (Hilton et al., 2013; Pan, 2023).

Together, these studies have provided valuable conceptual foundations for understanding mammalian cilium disassembly. Ongoing advances in molecular genetics, proteomics and imaging across the tree of life promise continued insights from unicellular and non-mammalian species.

## Box 2: Key open questions and challenges in cilia disassembly

### Contexts and modes governing disassembly

- How do pre-mitotic, differentiation-associated and stimulus-induced disassembly differ, and what are their functional roles in development and tissue homeostasis?
- Are there more stimuli that trigger cilia disassembly across physiologic and developmental contexts?
- How do different cell types and physiologic conditions determine whether cilia undergo gradual shortening, stochastic deciliation, internalization or shedding?

### Mechanisms and regulation

- How does regulation of microtubule PTMs contribute to cilia stability and disassembly, and how do PTMs modulate enzymes that sever or depolymerize microtubules?
- What are the mechanisms of ciliary membrane scission and resorption, and how do these processes dictate the fate of the ciliary membrane during disassembly?
- To what extent and by what mechanisms is the transition zone altered, removed or recycled during cilia disassembly?
- How do upstream cues such as mitogens, GPCR ligands, mechanical forces and stresses couple to specific downstream disassembly modules?
- How are disassembly mechanisms at the axoneme, ciliary membrane, centrosomal and cell body disassembly coordinated?

### Integration with ciliogenesis

- Are there positive feedback loops, ultrasensitive steps or mutual inhibitory motifs between ciliogenesis and disassembly pathways that are sufficient to generate switch-like or bistable control of ciliation?
- Do ciliated and non-ciliated states exhibit hysteresis – for example, do thresholds for cilium assembly and disassembly depend on a cell’s history?
- How do cell cycle, transcriptional and metabolic programs jointly shape the “ciliation landscape”, and how does cell state influence the permanence versus reversibility of disassembly?

### Disease relevance and therapeutic opportunities

- What additional diseases involve altered cilia disassembly, and how do such alterations contribute to pathogenesis?
- For diseases associated with multi-functional regulators that influence cilia disassembly, which disease features are due to altered ciliation versus non-ciliary functions?
- Can we find strategies to enhance or inhibit disassembly in a cell type-specific manner to correct pathologic changes in ciliation and provide therapeutic benefits?

### Technical challenges

- Can we develop and improve methods to track cilia disassembly *in vitro* and *in vivo* to observe physiologic modes of cilia disassembly as well as the consequences of dysregulated disassembly?
- Can we develop potent and specific tools for targeted manipulation of ciliation to rigorously test causal roles of ciliary alterations in disease ?
- How can systematic proteomic and functional genomic approaches be used to build a more complete inventory of cellular components that participate in cilia disassembly?

Continued innovation in imaging, proteomics and functional genomics should help overcome these challenges, enabling future work to define the roles of cilia disassembly in development and to uncover new mechanisms of disease.
